# Surgery for Active Infective Endocarditis on Mitral Valve: Anatomical, Surgical, and Disease Factors as Long-Term Outcome Modifiers

**DOI:** 10.3390/medicina60060879

**Published:** 2024-05-27

**Authors:** Paolo Berretta, Olimpia Bifulco, Suvitesh Luthra, Chiara Tessari, Anna Zingale, Emma Bergonzoni, Michele Galeazzi, Valentina Lombardi, Pietro Giorgio Malvindi, Gino Gerosa, Sunil Ohri, Marco Di Eusanio

**Affiliations:** 1 Cardiac Surgery Unit, Lancisi Cardiovascular Center, Ospedali Riuniti delle Marche, Polytechnic University of Marche, 60126 Ancona, Italy; 2 Cardiac Surgery Unit, Department of Cardiac, Thoracic, Vascular Sciences and Public Health, University of Padua, 35128 Padova, Italy; 3 Wessex Cardiothoracic Centre, Southampton General Hospital, University Hospital Southampton, Southampton SO16 6YD, UK

**Keywords:** mitral valve, infective endocarditis, long-term outcomes

## Abstract

*Background and Objectives*: Determinants of long-term outcomes after surgery for native mitral valve endocarditis have not been thoroughly investigated. The aim of this study was to assess anatomical, disease, and surgical risk factors for long-term mortality and need of reintervention, in patients undergoing mitral valve surgery for active endocarditis. *Materials and Methods*: Patients who underwent surgery for active native mitral valve endocarditis at three academic centres, between 2000 and 2022, were analysed. The primary outcome was long-term survival. The secondary outcome was the freedom from mitral reoperation. Survival curves were constructed with Kaplan–Meier methodology. Multivariable Cox regression was used to identify demographic, anatomical, disease, and surgical factors associated with late mortality and reoperation. *Results*: 335 consecutive patients with active mitral endocarditis were analysed. Two hundred and one patients (70.5%) had infection confined to the valve cusp whereas 89 (25.6%) had invasive disease extended to the annulus and surrounding tissues. Preoperative neurological events occurred at the diagnosis in 52 cases. Streptococci were the most common causative organisms followed by *Staphylococcus aureus*, Coagulase-negative *Staphylococcus*, and *Enterococcus*. Valve repair was performed in 108 patients (32.2%). Survival at 5 and 10 years was 70.1% and 59.2%, respectively. *Staphylococcus* emerged as an independent predictor of late mortality, along with age, chronic obstructive pulmonary disease, and previous cardiac surgery. Survival was considerably reduced in patients with *S. aureus* compared with those without (log rank *p* < 0.001). The type of surgery (repair vs. replacement) did not emerge as a risk factor for late mortality and reoperation. Seventeen patients underwent mitral reoperation during the follow-up. The 5- and 10-year freedom from reoperation was 94.7% and 91.8%, respectively. *Conclusions*: Active mitral valve endocarditis remains a life-threatening disease with impaired survival. While lesion characteristics influenced surgical decision-making and intraoperative management, their impact on long-term survival and freedom from reintervention appears to be moderated by other factors such as infecting pathogens and patient comorbidities.

## 1. Introduction

Despite recent advances in diagnoses and management, active native mitral valve (MV) infective endocarditis (IE) remains a life-threatening condition, associated with high mortality and morbidity rates [1,2,3,4].

In the setting of MV IE, clinical advantages of repair over replacement have not been clearly established as in degenerative mitral valve disease.

Patients’ preoperative condition and severity of tissue destruction might limit successful repair or increase the complexity of treatment; nevertheless, use of a prosthetic valve should be limited due to the risk of reinfection [5,6,7]. A timely surgical treatment has shown potential to improve clinical outcomes in appropriately selected patients [8]. However, navigating this field is particularly challenging due to the inherent heterogeneity in the existing literature.

Current evidence is limited to observational studies with heterogenous patient populations including both healed and active infections, native and prosthesis valve infective endocarditis, and cases involving single and multiple valves [9,10,11]. Moreover, the impact of anatomical presentations and the type of lesions, and the determinants of long-term outcomes, have not been thoroughly investigated [12,13]. The aim of this study was to examine the prognostic impact of anatomical, disease, and surgical risk factors for late mortality and need of reintervention, in patients undergoing native mitral valve surgery for active infective endocarditis at three referral surgical centres.

## 2. Materials and Methods

### 2.1. Study Design and Ethical Approval

This is a multicentre retrospective study on prospectively collected data. This study was approved by a local institutional review board (CERM 212813, 2023).

### 2.2. Population and Data Collection

All consecutive patients who underwent mitral valve surgery for isolated active IE at 3 referral centres, between 2000 and 2022, were enrolled. Patients with multiple valve endocarditis and prosthesis valve endocarditis were excluded. Preoperative, intraoperative, and postoperative data were retrieved from the heart valve infective endocarditis internal database of the Cardiac Surgery Unit at Lancisi Cardiovascular Centre in Ancona (Italy); the internal database of the Cardiac Surgery Unit, Wessex Cardiothoracic Centre, at the University Hospital Southampton in Southampton (United Kingdom); and the internal database of the Cardiac Surgery Unit at the Cardio-thoraco-vascular and Public Health Department, Padova, University Hospital. The patients were followed by the outpatients’ clinic and telephone calls. Patients’ follow-up was closed in March 2023. Mortality data were derived from the municipal administration records and mitral valve reintervention events were retrieved by cross-referencing the registry’s database. The primary outcome was long-term survival. The secondary outcome was the freedom from mitral valve reoperation.

### 2.3. IE Characterization and Management

The diagnosis of infective endocarditis was based on clinical suspicion supported by microbiological data and evidence of cardiac structure involvement provided by imaging techniques [14]. Infective endocarditis was managed through a multidisciplinary approach, involving cardiologists, radiologists, internal medicine, and infectious disease specialists. The aetiology of endocarditis was identified by microbiological laboratory cultures and serological testing. Three sets of blood cultures were performed before initiations of medical therapy. Causative pathogens were classified as follows: *Staphylococcus aureus*, Coagulase-negative *Staphylococcus*, *Enterococcus*, Streptococcus species, Gram-positive coccus, Fungal, Polymicrobial, and other. In all cases, a pathological examination of resected tissue was performed. A full-body computed tomography (CT) scan was performed in all patients. In patients with major cerebrovascular lesions observed on the CT scan, surgery was delayed by 4–6 weeks [14]. The characterization of the pathology, site, and type of valvular lesions (vegetation, leaflet perforation) was documented according to a transoesophageal echocardiogram and operative reports, and it estimates the local complication of IE. The involvement of the mitral valve annulus, commissures, or surrounding structures (abscess, false aneurysm, fistula) was classified as extended disease. All patients underwent mitral valve surgery before the completion of a full therapeutic course of antibiotics (the standard regimen of treatment was 4–6 weeks). Empiric therapy was started at the time of diagnosis and subsequently adjusted according to an antibiogram under the care of Microbiology. The choice of empirical therapy was influenced by previous antibiotic therapy, place of infection, local epidemiology, and possible antibiotic resistance. The decision for surgical treatment was established by the endocarditis team accounting for the urgency of patients’ clinical condition, comorbidities, perioperative risk, and potential benefit and prognosis. The indication for surgery included the prevention of an embolism, signs of uncontrolled infection, and heart failure. The goal of the surgical procedure was the radical removal of infected and necrotic tissue, combined with valve repair or replacement. Several techniques were used for mitral valve repair including the resection of infected tissue, resection and/or patch repair of mitral valve leaflets, and implantation of artificial chords, with or without ring annuloplasty. Mitral valve replacement was undertaken with stented bioprosthetic or mechanical valves, which were implanted using interrupted sutures with pledgets. The choice between a bioprosthetic or mechanical prosthesis was made according to patients’ characteristics, concomitant clinical status, and patient preference. The decision to repair or replace the valve was based on intraoperative evaluation and the surgeon’s judgment. All operations were performed through full sternotomy access.

### 2.4. Statistical Analysis

Continuous variables are expressed as means ± SDs or medians and interquartile ranges (IQRs), while categorical variables are presented as numbers and percentages. Student t or Mann–Whitney *U* tests and a chi-square test were used to compare continuous or categorial variables, respectively. In all cases, missing data were not defaulted to be negative, and denominators reflect only cases reported. Multivariable logistic regression was used to evaluate predictors of valve repair. The covariates included in the model were age, sex, previous cardiac surgery, chronic obstructive pulmonary disease, chronic kidney disease, urgent clinical status, type of valve lesion (vegetation; cusp tear; anterior, posterior, or bileaflet involvement; commissures; or annulus damage), and microorganisms. Survival curves were constructed with Kaplan–Meier methodology and compared with the log rank test. The multivariable association between the type of valve lesion (vegetation; cusp tear; anterior, posterior, or bileaflet involvement; extended disease), pathogens, and the study outcomes was assessed using a multivariable Cox regression analysis. The models were adjusted for potential confounders selected a priori based on their clinical significance that may directly influence the long-term results [age, sex, previous cardiac surgery, diabetes, smoking, chronic obstructive pulmonary disease, chronic kidney disease, peripheral arteriopathy, stroke, emergency, NYHA class III–IV, reduced left ventricular ejection fraction, type of surgery (repair vs. replacement), concomitant procedures (coronary artery bypass grafting, aortic valve replacement, and tricuspid valve surgery)].

The follow-up was 99.4% complete. *p*-Values < 0.05 were considered statistically significant. The analysis was generated using Statistical Package for Social Sciences version 29.0 (IBM SPSS Inc., Chicago, IL, USA).

## 3. Definitions

An emergent status was considered if the operation was performed within 24 h from hospital admission. Extended disease was classified as infective endocarditis with the involvement of the mitral valve annulus, commissures, or surrounding structures. In-hospital mortality was defined as all-cause mortality in the hospital stay after the mitral valve operation. Postoperative bleeding was considered surgical exploration for the purpose of controlling haemorrhage.

## 4. Results

### 4.1. Baseline Characteristics

During the study period, 335 patients underwent mitral valve surgery for isolated active IE. The median age of the study population was 64 (53–71) years, and 239 patients (71.3%) were male. Eighty-four (25.7%) patients were in NYHA III–IV. Left ventricle systolic disfunction (EF < 50%) was reported in 59 cases (18.3%). Previous stroke due to a septic embolism was observed in 52 patients (15.8%). Previous cardiac surgery occurred in 18 cases (5.5%). Sixty-nine mitral valve procedures (20.6%) were performed on an emergency basis. Table 1 details all patients’ characteristics.

### 4.2. Endocarditis Characteristics

Infective endocarditis involved the posterior leaflet in 13.3% (*n* = 41) of patients, the anterior leaflet in 22.7% (*n* = 70) of patients, and both leaflets in 26.2% (*n* = 81) of cases. Vegetations were found in 260 patients (84.1%) and extended disease occurred in 25.6% of cases (*n* = 89). Streptococcus was the most common causative pathogen (*n* = 127, 38%), followed by *Staphylococcus aureus* (*n* = 72, 22.7%), Coagulase-negative *Staphylococcus* (*n* = 23, 6.9%), and *Enterococcus* (*n* = 23, 6.9%). In 13.5% of cases (*n* = 45), no causative microorganism was found using the usual blood culture methods. Endocarditis characteristics are reported in Table 2.

### 4.3. Operative Data

Mitral valve replacement was performed in 227 patients (67.8%) and 108 patients (32.2%) received mitral valve repair. Primary leaflet resection was performed in 80 patients (14.9%), and the implantation of artificial chords was used in 15 patients (4.5%). Primary leaflet(s) resection and repair, the excision of vegetation(s), and patch repair were the most used techniques for mitral valve repair.

**Figure 1:**
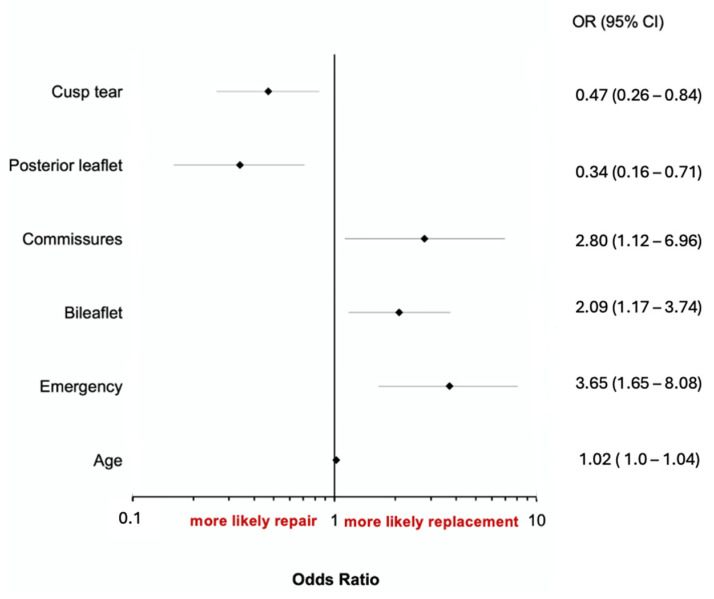
Predictors of mitral valve repair.

Posterior leaflet disease (OR: 0.34, 95%IC: 0.16–0.71, *p* = 0.04) and cusp tear (OR: 0.47, 95%IC: 0.26–0.84, *p* = 0.01) were associated with an increased likelihood of valve repair (Figure 1). Conversely, age (OR: 1.02, 95%IC: 1.0–1.04, *p* = 0.03) and urgent clinical status (OR: 3.65, 95%IC: 1.65–8.08, *p* = 0.001), as well as bileaflet (OR: 2.09, 96%IC: 1.17–3.74, *p* = 0.01) and commissure (OR: 2.80, 95%IC: 1.12–6.96, *p* = 0.03) involvement, emerged as independent predictors of a decreased probability of valve repair. In the replacement group, biological prostheses and mechanical prostheses were used, respectively, in 67% and in 33% of patients. The median time of cardiopulmonary bypass was 120 (88–157) minutes, and the median time of cross-clamp time was 92 (66.8–123) minutes.

### 4.4. Postoperative Early Outcomes

The overall in-hospital mortality rate was 8.1% (*n* = 27). Postoperative stroke occurred in 13 patients (4%). Renal replacement therapy was necessary in 14 patients (4.3%). Surgical revision due to postoperative bleeding was performed in 15 cases (4.6%). Operative data and in-hospital results are reported in Table 3.

### 4.5. Long-Term Outcomes

**Figure 2:**
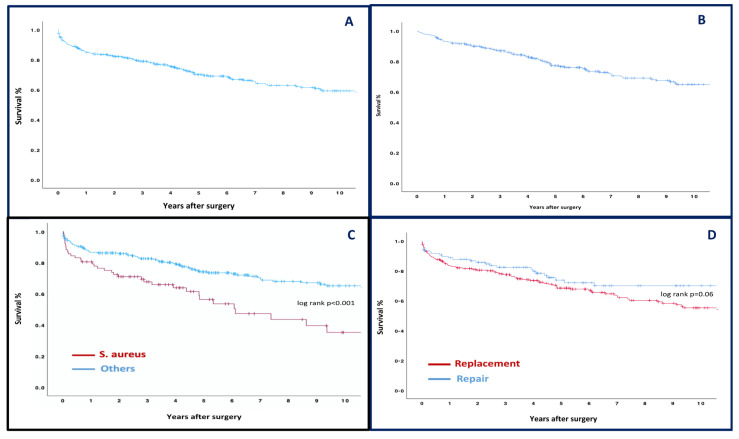
Kaplan–Meier analysis estimates of survival in all patients (panel **A**); landmark analysis of hospital survivors (panel **B**); and Kaplan–Meier analysis estimates of survival in patients with and without S. Aureus infection (panel **C**) and in patients who underwent MV repair or replacement (panel **D**).

**Figure 3:**
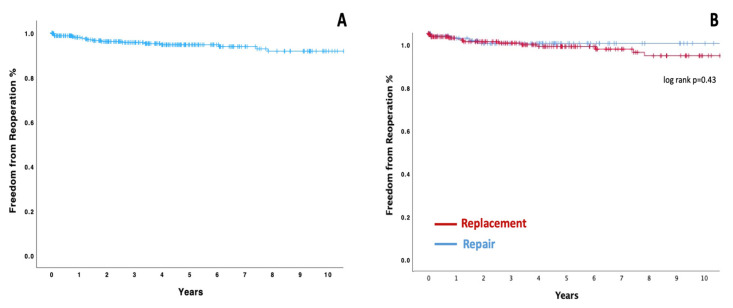
Kaplan–Meier analysis estimates of freedom from reoperation in all patients (panel **A**) and in patients who underwent MV repair or replacement (panel **B**).

One hundred and twenty-two patients died during FU. The overall 1-, 5-, and 10- year survival was 85%, 70.1%, and 59.2%, respectively (Figure 2—panel A). The landmark analysis of hospital survivors showed a 1-, 5-, and 10-year survival rate of 93.1%, 76.6%, and 64.8%, respectively (Figure 2—panel B). The multivariable analysis identified *Staphylococcus aureus* (HR: 2.66, 95%CI: 1.43–4.91) as an independent predictor of late mortality, along with age (HR: 1.04, 95%CI: 1.03–1.07), COPD (HR: 2.99, 95%CI: 1.60–5.60), and previous cardiac surgery (HR: 3.41, 95%CI: 1.21–9.61). All details are described in Table 4. The estimated survival rates at 1, 5, and 10 years for patients with and without S. Aureus were 80.5%, 56.3%, and 35.2% and 87.4%, 74.1%, and 65.1%, respectively (log rank *p* < 0.001) (Figure 2—panel C). Survival probabilities were similar considering patients after mitral valve repair and replacement (Figure 2—panel D). Seventeen patients underwent mitral valve reoperation during the follow-up. The overall 1-, 5-, and 10-year freedom from reoperation was 98%, 94.7%, and 91.8%, respectively (Figure 3—panel A). Ten-year freedom from reoperation was 90.2% vs. 95.6%, respectively, in replacement and repair groups (long rank *p* = 0.43) (Figure 3—panel B). Cox regression failed to identify any independent predictors for mitral reintervention.

## 5. Discussion

Results after mitral valve surgery for active infective endocarditis have shown limited improvement, over the past years. While timely surgical treatment has shown promise in improving operative outcomes, current evidence is limited to observational studies with heterogeneous patient populations, making it difficult to evaluate determinants of a long-term prognosis [12,13]. Clinical trials revealed the influence of an initial poor clinical condition and comorbidities on short-term results; yet, the long-term implications of anatomical, clinical, and disease-related factors remain uncertain [1,2,3,4]. In such complex scenarios, comprehensive risk stratification becomes crucial to identify patients with adverse prognostic profiles, facilitating tailored treatment strategies and timely surgical interventions [15,16,17,18]. In the present multicentric study, we assessed short- and long-term outcomes in patients with isolated active endocarditis on the native mitral valve. By focusing exclusively on this IE patient subset, our study may offer more targeted and clinically relevant insights into the long-term prognosis and risk factors associated with adverse outcomes. Our findings confirmed the significant mortality and morbidity burden associated with this condition, with an in-hospital mortality rate of 8.1% and a stroke rate of 4%. The long-term analysis revealed a persistent risk of mortality associated with mitral infective endocarditis, with the overall survival rate declining from 85% at one year to 59.2% at ten years. Active native mitral IE presented a wide spectrum of lesions, ranging from isolated single tear or vegetation requiring simple valve repair to extended disease requiring complex valve reconstruction or valve replacement. A bileaflet lesion was observed in 26.2% of cases, while extended disease involving the valve annulus and commissures was found in more than a quarter of patients. While the type, location, and extension of anatomical lesions were found to influence the type of treatment and operative results [19,20,21,22,23,24,25,26], the present analysis revealed no significant impact on long-term outcomes within our study cohort. These findings suggest the multifactorial nature of disease progression and support the importance of comprehensive risk assessment in such high-risk patients. Notably, the type of surgery (repair vs. replacement) did not emerge as a significant predictor of late mortality or the need for reoperation. The role of valve repair in mitral valve endocarditis was supported by physiological and clinical benefits provided by the preservation of the native mitral apparatus and the prevention of the inherent risks of prosthetic valves. Mitral valve repair was determined based on patient comorbidities and the anatomy of the lesion. Repair was more commonly performed in patients with an isolated posterior single leaflet lesion or cusp tear. Conversely, a bileaflet lesion and commissural involvement were more frequently addressed with valve replacement. This approach yielded a repair rate of 32.2%, which, nevertheless, exceeds that reported in previous series, and provided an overall acceptable rate of late mitral reoperation [19,20,21,22,23,24,25,26]. These results were likely related to a careful patient selection and to the high experience in mitral valve surgery of centres and surgeons included in this study. The role of lesion localization and local invasiveness in the success of mitral valve repair has been well highlighted by Miura et al. [27]. Considering a cohort of patients with a high rate of mitral repair (71.6% of the overall population with mitral endocarditis), they reported satisfactory durability at 10 years in patients with posterior leaflet involvement or a limited lesion of the anterior leaflet without annular invasion. The patching and leaflet extension of the anterior mitral leaflet can increase the chance of preserving the mitral apparatus, but the risk of reoperation is still high [28,29]. Our findings confirmed that the surgical treatment in patients with active infective endocarditis has to be based on an individualized strategy tailored to both patient-specific factors and disease characteristics [26].

The increasing aging population at risk of infective endocarditis and the incidence of nosocomial procedures have shifted the microbiology of infective endocarditis [30]. The emergence of healthcare-associated infective endocarditis has led to an increase in the prevalence of *Staphylococcus aureus*, usually responsible for acute and destructive endocarditis [14]. Fowler et al. [31] analysed data from the International Collaboration on Endocarditis, accounting for 1779 patients with infective endocarditis in a prospective observational study in 39 medical centres of 16 countries. *S. aureus* was the most common identified pathogen (31.4%) and patients with *S. aureus* IE were more likely to have healthcare-associated infection. The virulent nature of *S. aureus*, characterized by large vegetations and paravalvular abscesses, has been associated with impaired long-term results [31,32,33]. After internalization by endothelial cells in vitro, *S. aureus* evokes a potent proinflammatory chemokine response, including an increased expression of IL-6, IL-8, and monocyte chemotactic peptide. This molecular mechanism together with the action to produce biofilms explain the clinical impact of *S. aureus* IE and the resistance to antibiotic therapy.

In our study, patients infected with this pathogen experienced a substantially reduced survival rate compared to those without (65.1% vs. 35.2% at 10 years). This confirms the virulence and clinical significance of *Staphylococcus aureus* and supports the need for targeted therapeutic interventions and vigilant postoperative management in affected individuals.

## 6. Limitations

This study is a retrospective analysis on prospectively collected data from three surgical centres with limitations inherent to study design. In our analysis, failure to find risk factors for reoperations could be attributed to the relatively small number of patients enrolled and events that occurred. Another limitation of this study is that the reoperation events for reinfection were not distinguished from the overall reintervention rate. In addition, no echocardiographic data at the follow-up and no data on the causes of late mortality were available; reoperation is related to a clinical decision, and it is not the ideal marker to weigh the failure of a valve/prosthesis over time.

## 7. Conclusions

This multicentric study provides insights into the determinants of long-term outcomes in patients undergoing mitral valve surgery for active native endocarditis. While lesion characteristics influenced surgical decision-making and intraoperative management, their impact on long-term survival and freedom from reintervention appears to be moderated by other factors such as infecting pathogens and patient comorbidities. Further research is warranted to identify novel therapeutic strategies to improve late outcomes in this high-risk patient population.

## Figures and Tables

**Table 1:** Demographics.

	*n* = 335 *N* (%)
Male	239 (71.3)
Age, years (median IQR)	64 (53–71)
BMI, kg/m^2^ (median IQR)	24.1 (21.3–27)
NYHA III–IV	84 (25.7)
Hypertension	115 (34.7)
Diabetes	54 (16.3)
Smoking	128 (38.2)
CKD (creatinine > 2 mg)	32 (9.6)
COPD	43 (13)
Previous stroke	52 (15.8)
Peripheral arteriopathy	11 (4.1)
Previous cardiac surgery	18 (5.5)
Preoperative mechanical ventilation	17 (6.3)
Preoperative IABP	1 (1.3)
LVEF < 50%	59 (18.3)
Urgent/emergent status	69 (20.6)

BMI: body mass index. CKD: chronic kidney disease. COPD: chronic obstructive pulmonary disease. IQR: interquartile range. LVEF: left ventricular ejection fraction. NYHA: New York Heart Association.

**Table 2:** Type and site of IE lesions, and microbiological data.

	*n* = 335 *N* (%)
Pathology	
Site	
Annulus	33 (10.7)
Posterior leaflet	100 (32.4)
Anterior leaflet	70 (22.7)
Bileaflet	81 (26.2)
Commissures	66 (21.4)
Lesions	
Vegetations	260 (84.1)
Cusp tear	134 (45.6)
Extended disease	89 (25.6)
Microorganism	
*Staphylococcus aureus*	75 (22.7)
Coagulase-negative *Staphylococcus*	23 (6.9)
Enterococcus	23 (6.9)
Streptococcus species	127 (38.4)
Gram-positive coccus	15 (4.5)
Fungi	-
Polymicrobial	10 (3)
Culture-negative endocarditis	45 (13.4)
Other	13 (3.9)

**Table 3:** Operative data and early postoperative outcomes.

	*n* = 335 *N* (%)
Procedure	
Mitral valve repair	108 (32.2)
Anterior leaflet lesion	25 (23.1)
Posterior leaflet lesion	55 (50.9)
Bileaflet lesion	17 (15.7)
Extended disease	17 (15.7)
Mitral valve replacement	227 (67.8)
Anterior leaflet lesion	45 (19.8)
Posterior leaflet lesion	45 (19.8)
Bileaflet lesion	64 (28.2)
Extended disease	72 (31.7)
Type of mitral valve repair	
Ring implantation	80 (23.9)
Primary leaflet(s) resection/repair	50 (14.9)
Shaving leaflet	16 (4.8)
Implantation of artificial chords	15 (4.5)
Leaflet(s) repair with patch	13 (3.8)
Type of prosthesis	
Biological	152/227 (67)
Mechanical	75/227 (33)
Concomitant procedure	
Aortic valve replacement	102 (30.4)
Tricuspid valve repair	26 (7.8)
CABG	36 (10.7)
CPB time (minutes), median (IQR)	120 (88–157)
X-Clamp time (minutes), median (IQR)	92 (66.8–123)
Postoperative data	
In-hospital mortality	27 (8.1)
Stroke	13 (4)
Dialysis	14 (4.3)
Bleeding	15 (4.6)
Deep wound complication	4 (2.3)
Hospital stay (days)	19 (8–22)

CABG: coronary artery bypass graft. CPB: cardiopulmonary bypass. IQR: interquartile range.

**Table 4:** Predictors for reduced survival (backward Cox regression analysis).

	HR	95% CI	*p*-Value
Age	1.04	1.03; 1.07	<0.001
Previous cardiac surgery	3.41	1.21; 9.61	0.02
COPD	2.99	1.60; 5.60	0.001
Vegetations	2.17	0.87; 5.18	0.07
Arteriopathy	3.46	1.01; 12.7	0.05
Pathogens			
*Streptococcus* species (ref.)	*—*	*—*	*—*
Coagulase-negative Staphylococcus	0.81	0.30; 2.21	0.8
*Enterococcus*	0.85	0.24; 3.04	0.8
*Gram-positive coccus*	1.89	0.41; 8.67	0.4
Polymicrobial	2.76	0.62; 12.18	0.2
Other	1.03	0.51; 2.08	0.9
*Staphylococcus aureus*	2.66	1.43; 4.91	0.002
Type of surgery (repair vs. replacement)	1.27	0.75; 2.14	0.37

CI = confidence interval. COPD: chronic obstructive pulmonary disease. HR = hazard ratio.

## Data Availability

The data underlying this article will be shared on reasonable request to the corresponding author.
